# Dietary and herbal supplements for weight loss: assessing the quality of patient information online

**DOI:** 10.1186/s12937-021-00729-x

**Published:** 2021-07-27

**Authors:** Jeremy Y. Ng, Saad Ahmed, Catherine Jiayi Zhang

**Affiliations:** Department of Health Research Methods, Evidence, and Impact, Faculty of Health Sciences, McMaster University, Michael G. DeGroote Centre for Learning and Discovery, Room 2112, 1280 Main Street West, Hamilton, ON L8S 4K1 Canada

**Keywords:** Weight loss, Dietary and herbal supplements, Quality of information, Internet, Website, Consumer health information, Information assessment, DISCERN

## Abstract

**Background:**

Given the high prevalence of dietary and herbal supplement (DHS) use in tandem with the growing ease of internet access, patients commonly search online for consumer health information about these products. One common reason for DHSs use includes weight loss. Healthcare providers need to be aware of the quality of online information about DHSs for weight loss so they can adequately counsel their patients and provide them with guidance surrounding the identification of high-quality information resources. This study aimed to assess the quality of online DHSs consumer health information for weight loss that a “typical” patient might access online.

**Methods:**

Six search terms were used to generate the first 20 websites on the Google search engine in four countries: Australia, Canada, the United Kingdom, and the United States (n = 480 websites). After applying exclusion criteria, eligible websites were quality assessed using the DISCERN instrument. This tool is comprised of 16 questions, each evaluated on a 5-point scale. The averages and standard deviations for each DISCERN instrument item, in addition to overall summed scores between 15 and 75 were calculated.

**Results:**

Across 87 eligible websites, the mean summed score was 44.80 (SD = 11.53), while the mean overall DISCERN score of each website was 2.72 (SD = 0.99). In general, websites detailed and achieved their specified aims and described treatment benefits. However, most websites failed to describe the impact of treatment on overall quality of life and the impact of a no treatment option. The highest-scoring websites were largely government or health portal websites, while the lowest-scoring websites were largely commercial in nature.

**Conclusion:**

High variability in DISCERN instrument scores was found across all websites assessed. Healthcare providers should be aware of the fact that their patients may be accessing misinformation online surrounding the use of DHSs for weight loss. Therefore, it is important for healthcare providers to ensure that they are providing their patients with guidance on how to identify high-quality resources online, in order that safe, effective, and evidence-based decisions are made surrounding the use of DHSs for weight loss.

**Supplementary Information:**

The online version contains supplementary material available at 10.1186/s12937-021-00729-x.

## Background

In 2018, an estimated 42.4% of American adults and 18.5% of American adolescents were classified as obese, which is defined as having a body mass index greater than 30 kg/m^2^ [1, 2]. Significant medical and psychosocial impacts are associated with obesity, including difficulty in making major dietary and lifestyle improvements [3]. Thus, patients often turn to dietary and herbal supplements (DHSs) to help achieve their weight loss goals [4]. According to the National Institutes of Health, DHSs are oral products that supplement the diet and contain ingredients such as vitamins, minerals, herbs, botanicals, amino acids, enzymes, and extracts [5]. The use of DHSs for weight loss is widespread, with reports indicating that over 15% of American adults have, at some point, used DHSs for weight loss [6]. In fact, the sales revenue of weight loss supplements is reported to be the fastest growing segment of the dietary supplement industry, with Americans spending over 2 billion dollars in 2015 [7]. These supplements are widely marketed and readily available without a prescription at pharmacies, retail outlets, health food stores, and online retailers [8]. Many of these DHSs are marketed with claims of increasing satiety, metabolism, lipolysis, and thermogenesis while reducing appetite, body fat, and overall weight [9]. Ingredients commonly found in DHSs for weight loss include green tea, chromium picolinate, ephedra, ginger root (*Zingiber officinale*), guarana (*Paullinia cupana*), caffeine, hydroxycitric acid, Siberian ginseng (*Eleutherococcus senticosus*), cayenne (*Capsicum annuum*), and bitter orange (*Citrus aurantium*) [10]. In a 2019 study of the nine most commonly advertised weight loss supplements on Google and Amazon, it was suggested that many weight loss supplements are associated with the potential for physical harm, with side-effects as severe as liver and kidney failure being observed [10]. Thus, patients need scientifically accurate information about safety and efficacy to help guide their choices about weight loss supplements.

Information about DHSs for weight loss is widely available online. According to the Pew Research Center, 90% of the American population has internet access, with the offline population gradually declining from 48% of the total population in 2000 to just 10% in 2019 [11]. Due to this increase in accessibility, consumer health information is becoming increasingly sought after by patients browsing the the internet. A study found that 1 in 3 American adults use the internet to learn about a health concern [12]. Moreover, the amount of people in the United Kingdom who access the internet for health-related information increased from 24% in 2008 to 60% in 2020 [13]. A problem that has arisen from the minimal regulation and standardization of internet resources is the infrequent assessment of online health information quality [14]. An abundance of incomplete and inaccurate information is prevalent online, with millions of accessible resources accumulating daily [14]. Consumers can easily find information about DHSs on the internet, but the veracity of this information is often questionable [15]. Given the high incidence of DHS use and ease of internet access, consulting web-based resources may lead patients to replace or decrease conventional medication use in favour of DHSs [16].

To our knowledge, few studies over the past 5 years have evaluated the quality of online consumer health information about DHSs for weight loss that a “typical” patient might access. Therefore, the purpose of this study is to assess the quality of web-based information related to DHSs for weight loss based on the current online landscape, and thereby fill this void in the literature.

## Methods

### Search strategy and screening

A cross-sectional survey of websites providing DHSs consumer health information for weight loss was conducted. Six search terms were used to generate the first 20 websites on Google search engines in four countries: Australia (google.com.au), Canada (google.ca), the United Kingdom (google.co.uk), and the United States (google.com). These search terms included: ‘dietary supplements for weight loss’, ‘herbal medicine for weight loss’, ‘herbs for weight loss’, ‘natural health products for weight loss’, ‘natural products for weight loss’, and ‘supplements for weight loss’. Search terms were generated based on terms commonly-used to refer to DHSs [8, 17]. Searches involved simple words to simulate the information-seeking behaviour of “typical” patients with limited medical knowledge. It has been found that websites listed on the first page of a Google search generate 92% of all traffic. When moving to page two, traffic drops by 95%, then by 78% and 58% for the subsequent pages [18]. Thus, only the first two pages of search results were reviewed in this study. Searches were completed using only the Google search engine; s of August 2020, Google takes approximately 92% of the search engine market share worldwide, therefore this decision was reflective of where most patients would seek and access health information [19]. Searches were conducted using the Google Chrome browser in Incognito mode to prevent prior search history and cached information from affecting search results. All searches were completed on the same day to ensure consistency. This search strategy was designed by JYN and carried out by SA.

### Eligibility criteria

SA and CJZ reviewed the search results, and duplicate websites across searches were removed. Websites were screened for eligibility and included if they contained at least one webpage that detailed consumer health information related to DHSs for weight loss. Moreover, Wikipedia articles, webpages with only videos (e.g. YouTube), webpages from the same website, Amazon links, invalid addresses, peer-reviewed articles, non-English language websites, eBooks, forums, advertised and sponsored links, and websites that require paid membership to access relevant information were excluded from the study.

### Data extraction and website quality assessment

The DISCERN instrument is a standardized quality index of written consumer health information [20]. It was developed with the input of an expert panel, health information providers, and patients in collaboration with the National Health Service, British Library, and Oxford Research and Development Programme. This tool is comprised of a series of questions evaluated on a 5-point scale ranging from “no” to “yes.” A score of 1 represents a definite “no” which implies the quality criterion has not been fulfilled at all. A rating between 2 and 4 suggests that the website partially meets the criterion in question, with the specific rating depending on the extent of the website’s shortcomings. A score of 5 represents a definite “yes” which indicates the quality criterion has been completely fulfilled [20]. The DISCERN instrument questions are divided into three categories: the reliability of the publication, the specific details of the information about treatment choices, and the overall quality rating of the source of information. It is important to note that DISCERN instrument cannot be used to assess the accuracy of scientific evidence, as this would necessitate checking the information against other sources. Rather, the DISCERN instrument is used to assess the reliability of the website as a source for consumer health information. Furthermore, the DISCERN instrument cannot be used to assess the presentation of information (readability, graphics, layout, etc.) as many assessment tools already exist for this purpose. Instead, the DISCERN instrument fills a gap by examining what consumer health information is being provided, rather than how it is being provided. Through 15 quality criteria, the DISCERN instrument helps pinpoint common causes of inaccurate or unreliable information such as bias. Additional details about the DISCERN instrument can be found at: www.discern.org.uk/discern_instrument.php.

Prior to conducting data extraction and quality assessing eligible websites using the DISCERN instrument, a pilot test designed by JYN was completed by two independent reviewers – SA and CJZ. In this pilot test, data pertaining to three separate websites was extracted and assessed using the DISCERN instrument; both SA and CJZ met with JYN to discuss the data extraction and the scoring of each DISCERN instrument item in detail to standardize its use. After completing the pilot test, SA and CJZ independently completed the data extraction and quality assessment of eligible websites using the DISCERN instrument. SA and CJZ then met with JYN to compare their scores and discuss discrepancies. The mean of the two reviewers’ scores for each of the first 15 DISCERN instrument items was calculated, which yielded an overall summed DISCERN instrument score between 15 and 75 for each website. Next, the means and standard deviations for each DISCERN instrument item, as well as the mean and standard deviation across all 16 items were calculated and assessed.

SA and CJZ extracted the following data: website URL, website type, type(s) of DHSs discussed, type(s) of non-DHS therapies discussed, whether websites appeared in more than one search across different search terms and/or regions, and scores for the sixteen DISCERN instrument questions. Unique URLs that led to the same website were collapsed into one search result for the purpose of quality assessment using the DISCERN instrument. Thus, assessment criteria were applied to each full website, not just a single webpage.

## Results

### Search results

**Fig. 1:**
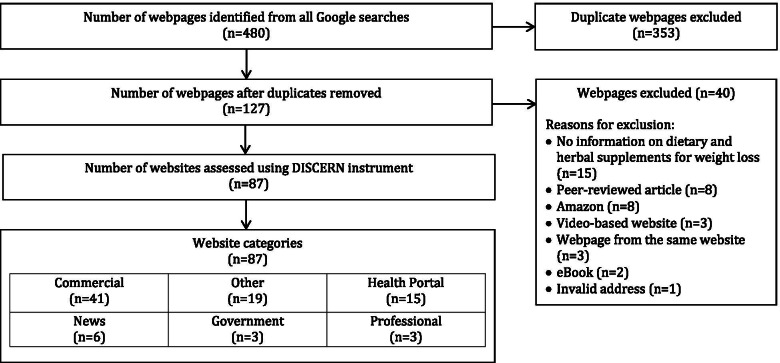
Web information search strategy and assessment flowchart

Of the 480 websites retrieved across all searches, 353 duplicates were removed. Of the remaining 127 websites, 40 websites were excluded for the following reasons: had no information on DHSs for weight loss (n = 15), peer-reviewed article (n = 8), Amazon links (n = 8), webpages with only videos (n = 3), webpages from the same website (n = 3), eBook website (n = 2), and invalid address (n = 1). The 87 websites that remained were deemed eligible for assessment. This process is depicted in Fig. 1.

### General characteristics of eligible websites

The 87 eligible websites were categorized into six groups. Commercial websites sell products or a service, such as GNC’s website (https://www.gnc.com/). Government websites, which are overseen by an official governmental body, such as the Government of Canada website (https://www.canada.ca/). Health portal websites, which feature a site-wide search option for various health information, such as WebMD (https://www.webmd.com/). News websites are overseen by media outlets that distribute news, such as the Times of India website (https://timesofindia.indiatimes.com/). Professional websites are created by professional organizations, health practitioners, and health experts, such as the Cleveland Clinic website (https://my.clevelandclinic.org/). Lastly, the “other” category includes websites that do not fit in any of the previously mentioned categories. The most common category was commercial websites, with 41 results. Next was “other” with 19 websites, followed by health portal websites with 15 results. All eligible websites discussed at least one of the four categorizations of DHSs by the U.S. Food and Drug Administration (FDA): vitamin and mineral products, botanical and herbal products, amino acid products, and enzyme supplements [21]. Some common examples of these products found on commercial websites included ephedra, glucomannan, and 5-hydroxytryptophan. Seventy-five websites discussed only DHS therapies, while only 12 websites discussed non-DHSs therapies, such as surgical procedures and pharmaceutical medications. Some commonly discussed examples of these included bariatric surgery and orlistat. Fifty-five websites appeared in more than one search. General characteristics of eligible websites are displayed in Table 1.

**Table 1:** General Characteristics of Eligible Websites

Website Name	URL	Type(s) of DHSs Discussed	Type(s) of Non-DHS Therapies Discussed	Appeared in More Than One Search?
Commercial (*n* = 41)				
Aus Natural Care	https://www.ausnaturalcare.com.au/	Vitamin and mineral products, botanical/herbal products	None	No
Australian Sports Nutrition	https://www.australiansportsnutrition.com.au/	Vitamin and mineral products, botanical/herbal products, amino acid products, enzyme supplements	None	No
Bodybuilding	https://www.bodybuilding.com/	Vitamin and mineral products, botanical/herbal products, amino acid products, enzyme supplements	Pharmaceuticals (e.g. orlistat), surgical procedures (e.g. bariatric surgery)	Yes
Boots	https://www.boots.com/	Vitamin and mineral products, botanical/herbal products, enzyme supplements	Pharmaceuticals (e.g. orlistat)	No
Botanic Choice	https://www.botanicchoice.com/	Botanical/herbal products	None	No
Botanica Health	https://botanicahealth.com/	Botanical/herbal products	None	No
Brighter Day Foods	https://brighterdayfoods.com/	Botanical/herbal products	None	No
Bulk Nutrients	https://www.bulknutrients.com.au/	Vitamin and mineral products, botanical/herbal products, amino acid products, enzyme supplements	None	Yes
Bulk Powders	https://www.bulkpowders.co.uk/	Vitamin and mineral products, botanical/herbal products, amino acid products, enzyme supplements	None	Yes
Carusos Natural Health	https://carusosnaturalhealth.com.au/	Botanical/herbal products	None	No
Chemist Warehouse	https://www.chemistwarehouse.com.au/	Vitamin and mineral products, botanical/herbal products	None	Yes
CVS	https://www.cvs.com/	Vitamin and mineral products, botanical/herbal products, amino acid products, enzyme supplements	Pharmaceuticals (e.g. orlistat)	Yes
Elite Supps	https://www.elitesupps.com.au/	Vitamin and mineral products, botanical/herbal products, enzyme supplements	None	No
Feel Good Natural	https://feelgoodnatural.com/	Vitamin and mineral products, botanical/herbal products	None	Yes
Fusion Health	https://www.fusionhealth.com.au/	Vitamin and mineral products, botanical/herbal products	None	No
Global Healing	https://globalhealing.com/	Botanical/herbal products	None	Yes
GNC	https://www.gnc.com/	Vitamin and mineral products, botanical/herbal products, amino acid products, enzyme supplements	Pharmaceuticals (e.g. orlistat)	Yes
Go Healthy	https://gohealthy.co.nz/	Vitamin and mineral products, botanical/herbal products	None	No
Healthy Being	https://www.healthybeing.com.au/	Botanical/herbal products	None	Yes
Herbal Magic	https://www.herbalmagic.ca/	Botanical/herbal products	None	Yes
Holland and Barrett	https://www.hollandandbarrett.com/	Vitamin and mineral products, botanical/herbal products	None	Yes
Indigo Herbs	https://www.indigo-herbs.co.uk/	Botanical/herbal products	None	Yes
Legion Athletics	https://legionathletics.com/	Vitamin and mineral products, botanical/herbal products	None	Yes
Mind Body Green	https://www.mindbodygreen.com/	Botanical/herbal products	None	Yes
My Nutricentre	https://www.mynutricentre.com/	Vitamin and mineral products, botanical/herbal products	None	No
My Vitamins	https://www.myvitamins.com/	Vitamin and mineral products, botanical/herbal products, amino acid products, enzyme supplements	None	No
My Protein	https://www.myprotein.com/	Vitamin and mineral products, botanical/herbal products, amino acid products	None	Yes
Natural Grocers	https://www.naturalgrocers.com/	Botanical/herbal products	None	No
Nature's Best	https://www.naturesbest.co.uk/	Vitamin and mineral products, botanical/herbal products, amino acid products	None	No
Now Foods	https://www.nowfoods.com/	Vitamin and mineral products, botanical/herbal products, amino acid products, enzyme supplements	None	Yes
Nutrition House	https://www.nutritionhouse.com/	Vitamin and mineral products, botanical/herbal products, amino acid products, enzyme supplements	None	No
Nutrition Warehouse	https://www.nutritionwarehouse.com.au/	Vitamin and mineral products, botanical/herbal products, amino acid products, enzyme supplements	None	Yes
OpenFit	https://www.openfit.com/	Botanical/herbal products	None	Yes
PatchMD	https://www.patchmd.com/	Vitamin and mineral products, botanical/herbal products	None	No
Pharmacy Online	https://www.pharmacyonline.com.au/	Vitamin and mineral products, botanical/herbal products, amino acid products	None	No
PHD	https://www.phd.com/	Vitamin and mineral products, botanical/herbal products, amino acid products, enzyme supplements	None	No
Priceline	https://www.priceline.com.au/	Vitamin and mineral products, botanical/herbal products	None	No
Shoppers Drug Mart	https://www1.shoppersdrugmart.ca/	Vitamin and mineral products, botanical/herbal products, amino acid products, enzyme supplements	Pharmaceuticals (e.g. orlistat)	No
Supreme Natural Health	https://supremenaturalhealth.com.au/	Botanical/herbal products, enzyme supplements	None	No
The Natural Way	https://www.thenaturalway.com.au/	Botanical/herbal products	None	No
True Protein	https://www.trueprotein.com.au/	Botanical/herbal products, amino acid products	None	Yes
Other (*n* = 19)				
Active	https://www.active.com/	Botanical/herbal products	None	Yes
Body and Soul	https://www.bodyandsoul.com.au/	Vitamin and mineral products, botanical/herbal products, amino acid products	None	Yes
Choice	https://www.choice.com.au/	Vitamin and mineral products, botanical/herbal products, enzyme supplements	None	No
Craig Lewis Fitness	https://craiglewisfitness.com/	Botanical/herbal products	None	Yes
Digital Welt	https://www.digitalwelt.org/	Botanical/herbal products	None	Yes
Family Living Today	https://familylivingtoday.com/	Vitamin and mineral products, botanical/herbal products, enzyme supplements	None	No
Health	https://www.health.com/	Botanical/herbal products	None	Yes
LifeHack	https://www.lifehack.org/	Vitamin and mineral products, botanical/herbal products, enzyme supplements	None	Yes
Lose Weight Loss	http://www.loseweightloss.net/	Botanical/herbal products	None	No
Men's Journal	https://www.mensjournal.com/	Vitamin and mineral products, botanical/herbal products, amino acid products	None	Yes
National University of Health Sciences	https://blog.nuhs.edu/	Vitamin and mineral products, botanical/herbal products	None	Yes
Natural Foods Market Online	https://nfmonline.com/	Botanical/herbal products	None	No
Natural Health Courses	https://naturalhealthcourses.com/	Botanical/herbal products	None	No
Nutrain Ingredients	https://www.nutraingredients.com/	Botanical/herbal products	None	Yes
Prevention	https://www.prevention.com/	Botanical/herbal products	None	Yes
Remedy Review	https://www.remedyreview.com/	Botanical/herbal products	None	Yes
Style Craze	https://www.stylecraze.com/	Botanical/herbal products	None	Yes
The Cut	https://www.thecut.com/	Botanical/herbal products	None	Yes
Women's Health Magazine	https://www.womenshealthmag.com/uk/	Vitamin and mineral products, botanical/herbal products, amino acid products	None	Yes
Health Portal (*n* = 15)				
Body Nutrition	https://bodynutrition.org/	Vitamin and mineral products, botanical/herbal products, amino acid products, enzyme supplements	None	Yes
Consumer Health Day	https://consumer.healthday.com/	Botanical/herbal products	None	Yes
Drugs	https://www.drugs.com/	Vitamin and mineral products, botanical/herbal products, enzyme supplements	Pharmaceuticals (e.g. orlistat)	No
Empower Your Health	https://www.empoweryourhealth.org/	Vitamin and mineral products, botanical/herbal products, enzyme supplements	None	Yes
Examine	https://examine.com/	Vitamin and mineral products, botanical/herbal products, enzyme supplements	None	Yes
Future's Recovery Healthcare	https://futuresrecoveryhealthcare.com/	Vitamin and mineral products, botanical/herbal products, enzyme supplements	None	No
Healthline	https://www.healthline.com/	Vitamin and mineral products, botanical/herbal products, enzyme supplements	None	Yes
Mayo Clinic	https://www.mayoclinic.org/	Vitamin and mineral products, botanical/herbal products, amino acid products, enzyme supplements	Pharmaceuticals (e.g. orlistat), surgical procedures (e.g. bariatric surgery)	Yes
Medicine Net	https://www.medicinenet.com/	Vitamin and mineral products, botanical/herbal products	None	Yes
Medscape	https://www.medscape.com/	Vitamin and mineral products, botanical/herbal products, enzyme supplements	None	Yes
NDTV	https://doctor.ndtv.com/	Botanical/herbal products	None	Yes
RenueRX	https://renuerx.com/	Vitamin and mineral products, botanical/herbal products	None	Yes
The Healthy	https://www.thehealthy.com/	Vitamin and mineral products, botanical/herbal products, amino acid products	None	Yes
Very Well Fit	https://www.verywellfit.com/	Vitamin and mineral products, botanical/herbal products, amino acid products	None	Yes
WebMD	https://www.webmd.com/	Vitamin and mineral products, botanical/herbal products, amino acid products, enzyme supplements	Pharmaceuticals (e.g. orlistat), surgical procedures (e.g. bariatric surgery)	Yes
News (*n* = 6)				
Coach Nine	https://coach.nine.com.au/	Vitamin and mineral products, botanical/herbal products	None	No
Express	https://www.express.co.uk/	Vitamin and mineral products, botanical/herbal products	None	No
Insider	https://www.insider.com/	Vitamin and mineral products, botanical/herbal products, enzyme supplements	None	Yes
Medical News Today	https://www.medicalnewstoday.com/	Vitamin and mineral products, botanical/herbal products, enzyme supplements	None	Yes
Shape	https://www.shape.com/	Botanical/herbal products	None	Yes
Times of India	https://timesofindia.indiatimes.com/	Botanical/herbal products	None	Yes
Government (*n* = 3)				
Government of Canada	https://www.canada.ca/	Vitamin and mineral products, botanical/herbal products, amino acid products, enzyme supplements	Pharmaceuticals (e.g. orlistat), surgical procedures (e.g. bariatric surgery)	No
Medline Plus	https://medlineplus.gov/	Vitamin and mineral products, botanical/herbal products, enzyme supplements	Pharmaceuticals (e.g. orlistat), surgical procedures (e.g. bariatric surgery)	Yes
NIH Office of Dietary Supplements	https://ods.od.nih.gov/	Vitamin and mineral products, botanical/herbal products, amino acid products, enzyme supplements	Pharmaceuticals (e.g. orlistat), surgical procedures (e.g. bariatric surgery)	Yes
Professional (*n* = 3)				
Cleveland Clinic	https://my.clevelandclinic.org/	Botanical/herbal products	Pharmaceuticals (e.g. orlistat), surgical procedures (e.g. bariatric surgery)	Yes
Draxe	https://draxe.com/	Vitamin and mineral products, botanical/herbal products, amino acid products, enzyme supplements	None	Yes
Wexner Medical	https://wexnermedical.osu.edu/	Vitamin and mineral products, botanical/herbal products, amino acid products	None	Yes

### DISCERN instrument ratings

The mean total DISCERN instrument score across all assessed websites was 44.80 (SD = 11.53) out of a maximum of 75. The mean overall DISCERN instrument score (Question 16) of each individual website was 2.72 (SD = 0.99). Large variability was present, with individual overall scores ranging from 1.5 to 5. Question 1, 2, and 10 of the DISCERN instrument had the highest mean total scores of 4.41, 4.56, and 4.82, respectively. Question 1 asks whether the aims are clear, question 2 asks whether the website achieves its aims, and Question 10 asks whether the website describes the benefits of each treatment. Question 12 and 13 of the DISCERN instrument had the lowest mean total scores of 1.26 and 1.51, respectively. Question 12 asks whether the website describes what would happen if no treatment is used, and Question 13 asks whether the website describes how the treatment choices affect overall quality of life. The three highest scoring websites were “Very Well Fit” (67.50), “NIH Office of Dietary Supplements” (66.50), and “Mayo Clinic” (66.50). The three lowest scoring websites were “Elite Supps” (28.00), “Supreme Natural Health” (29.00), and “Healthy Being” (29.00). A summary of DISCERN instrument ratings by website category is presented in Table 2. The DISCERN instrument scores for each individual website included and assessed in this study is available in Supplementary File 1.

**Table 2:** Summary of DISCERN Instrument ratings by website category

**Section**		**SECTION 1 Is the publication reliable?**								**SECTION 2 How good is the quality of information on treatment choices?**							**SECTION 3 Overall Rating of Publication**		
**DISCERN Item**		**1. Are the aims clear?**	**2. Does it achieve its aims?**	**3. Is it relevant?**	**4. Is it clear what sources of information were used to compile the publication (other than the author or producer)?**	**5. Is it clear when the information used or reported in the publication was produced?**	**6. Is it balanced and unbiased?**	**7. Does it provide details of additional sources of support and information?**	**8. Does it refer to areas of uncertainty?**	**9. Does it describe how each treatment works?**	**10.Does it describe the benefits of each treatment?**	**11. Does it describe the risks of each treatment?**	**12. Does it describe what would happen if no treatment is used?**	**13. Does it describe how the treatment choices affect overall quality of life?**	**14. Is it clear that there may be more than one possible treatment choice?**	**15. Does it provide support for shared decision-making?**	**16. Based on the answers to all of the above questions, rate the overall quality of the publication as a source of information about treatment choices**	**Standard Deviation of Overall Score (Q16)**	**DISCERN Score (Sums of Q1-Q15)**
**Commercial** **(n = 41)**	Means	4.35	4.40	3.87	1.87	1.98	1.94	2.32	1.48	2.44	4.82	1.61	1.04	1.16	2.18	2.55	2.17	0.42	37.99
	Standard Deviations	0.78	0.72	0.71	1.25	0.92	0.90	1.71	0.92	1.03	0.37	0.77	0.13	0.36	0.93	1.25	0.77	0.35	7.86
**Other** **(n = 19)**	Means	4.50	4.53	3.55	2.61	2.74	3.08	2.45	2.05	2.03	4.79	2.21	1.26	1.40	3.21	2.82	2.66	0.49	43.21
	Standard Deviations	0.91	0.68	0.72	1.13	0.89	1.25	1.15	1.60	0.94	0.56	1.33	0.92	0.49	1.10	0.99	0.67	0.34	7.50
**Health Portal** **(n = 15)**	Means	4.73	4.97	4.40	3.13	4.00	4.13	3.73	4.23	3.17	4.87	4.03	1.30	2.23	4.80	4.27	3.77	0.43	58.00
	Standard Deviations	0.46	0.13	0.57	1.42	1.30	1.11	1.45	1.31	0.96	0.40	1.23	0.68	1.15	0.65	1.16	0.78	0.36	8.73
**News** **(n = 6)**	Means	3.33	4.25	4.00	3.08	2.92	4.25	2.58	3.58	2.08	4.67	3.17	1.50	1.42	3.83	2.58	3.08	0.59	47.25
	Standard Deviations	0.41	0.82	0.77	1.16	1.16	0.99	1.28	2.01	0.49	0.61	1.72	0.55	0.20	1.63	1.86	1.11	0.29	10.61
**Government** **(n = 3)**	Means	5.00	5.00	4.83	3.00	4.00	4.33	4.00	5.00	3.17	5.00	4.83	2.33	3.83	5.00	5.00	4.17	0.71	64.33
	Standard Deviations	0.00	0.00	0.29	1.80	1.32	1.16	1.73	0.00	1.26	0.00	0.29	2.31	2.02	0.00	0.00	0.58	0.00	2.57
**Professional** **(n = 3)**	Means	4.50	5.00	3.83	3.00	3.50	4.17	3.67	4.17	3.50	5.00	4.17	2.67	1.33	4.17	4.83	3.17	0.24	57.50
	Standard Deviations	0.87	0.00	1.04	0.50	1.32	0.58	1.26	1.44	0.00	0.00	1.44	2.02	0.29	1.44	0.29	0.29	0.41	6.56
**TOTAL Means**		4.41	4.56	3.93	2.41	2.68	2.89	2.71	2.44	2.51	4.82	2.47	1.26	1.51	3.14	3.07	2.72	0.45	44.80
**TOTAL Standard Deviations**		0.79	0.66	0.75	1.33	1.27	1.40	1.60	1.72	1.03	0.42	1.50	0.80	0.88	1.43	1.42	0.99	0.34	11.53

*Note*: DISCERN item scores range from 1 to 5. A score of 1 implies that the quality criterion has not been fulfilled at all, a score between 2 and 4 indicates partial fulfilment, and a score of 5 indicates that the quality criterion has been completely fulfilled. Overall summed DISCERN scores range from 15 to 75 and represent the overall quality of a website’s DHSs consumer health information for weight loss

### Trends identified across resources assessed

#### Well-described benefits, and clearly outlined and achieved website aims

Question 10 (whether the website describes the benefits of each treatment) had the highest mean total score across all DISCERN instrument items, with 82 websites scoring a 4.5 or above. Similarly high scores were not observed for Question 11 (whether the website describes the risks of each treatment), which was especially pronounced among commercial websites.

Question 1 (whether the aims clear) and Question 2 (whether the website achieves its aims) had the next highest mean total scores across all DISCERN instrument items. Sixty-three and 71 websites scored a 4 or above for Question 1 and Question 2, respectively. Almost all eligible websites had an “About” section that included an overview indicating what each website was about, what it covered, and who was their intended audience. This resulted in consistently high scores for Question 1 and 2 across most websites.

#### Failure to detail impact of opting for no treatment and impact of treatment on overall quality of life

Question 12 (whether the website describes what would happen if no treatment is used) and Question 13 (whether the website describes how treatment choices affect overall quality of life) had the lowest mean total scores across all DISCERN instrument items. A score of 1.5 or below was rated for 81 websites for Question 12, and 75 websites for Question 13. Most websites discussed alternatives to DHSs but did not explicitly mention the effect of having no treatment. While the majority of websites included descriptions of risks and benefits, they failed to mention the overall impact of a treatment choice or choices on day-to-day living, leading to low scores on Question 13.

#### High-scoring government websites and health portals, and low-scoring commercial websites

It is interesting to note that 64% of the 25 highest-scoring websites were health portal or government websites, while 85% of the 25 lowest-scoring websites were commercial websites. Across all six websites categories, government websites had the highest mean DISCERN instrument score at 64.33, while commercial websites had the lowest mean DISCERN instrument score at 37.99. Government and health portal websites provided appropriate research citations, less biased assessments of DHSs for weight loss, and generally adequately comprehensive DHS information. In contrast, commercial websites frequently lacked information surrounding the risks and benefits of DHSs for weight loss, and provided more biased assessments of DHSs.

### Recommended websites for patients and consumers

Ten out of twelve of the highest scoring websites were health portals and government websites, all with total DISCERN instrument scores above 60. These websites commonly included an abundance of references to primary research literature, and links to additional sources of reading; in contrast; this was rarely present across commercial websites. Healthcare practitioners may find value in recommending these high-scoring websites to their patients. Additional details surrounding these recommended websites are depicted in Table 3.

**Table 3:** Recommended Websites for Patients and Consumers

Website name	URL	Summed DISCERN Score	Website Category	Target Audience	Frequency of Updates
Very Well Fit	https://www.verywellfit.com/	67.50	Health Portal	Healthcare providers, researchers, patients/public	Website states that they regularly update and fact-check to keep content "accurate, reliable, and trustworthy." Precise frequency of update is not known
NIH Office of Dietary Supplements	https://ods.od.nih.gov/	66.50	Government	Healthcare providers, researchers, patients/public	"Last updated" is present on all pages, with "update history" available as well. Precise frequency of updates and new content publication is not known
Mayo Clinic	https://www.mayoclinic.org/	66.50	Health Portal	Healthcare providers, researchers, patients/public	Mayo Clinic Proceedings are published monthly. Page update frequency is unknown
Drugs	https://www.drugs.com/	66.00	Health Portal	Healthcare providers, researchers, patients/public	"Last updated" is available on all pages, and is "Medically Reviewed" by professionals. Data is retrieved from sources including IBM Watson Micromedex, Cerner Multum™, Wolters Kluwer™, which are updated monthly, and other unnamed databases
Healthline	https://www.healthline.com/	65.50	Health Portal	Healthcare providers, researchers, patients/public	New content published weekly, unknown how often older content is reviewed/updated
WebMD	https://www.webmd.com/	65.50	Health Portal	Healthcare providers, researchers, patients/public	"Last reviewed" is provided on all pages. Unknown how often information is reviewed
Examine	https://examine.com/	65.00	Health Portal	Healthcare providers, researchers, patients/public	"Last updated" and "Reviewed by" provided on all pages. Unknown how precise update frequency is
Medline Plus	https://medlineplus.gov/	65.00	Government	Healthcare providers, researchers, patients/public	"Review date" provided, but frequency of review us unknown
Cleveland Clinic	https://my.clevelandclinic.org/	64.50	Professional	Healthcare providers, researchers, patients/public	"Last reviewed by a Cleveland Clinic medical professional on …" provided, but no precise update frequency known
Medical News Today	https://www.medicalnewstoday.com/	62.50	News	Healthcare providers, researchers, patients/public	"Last medically reviewed on…" provided, but no precise update frequency known. Daily content published
Government of Canada	https://www.canada.ca/	61.50	Government	Healthcare providers, researchers, patients/public	"Date modified:" provided, but no precise update frequency known
Medscape	https://www.medscape.com/	61.00	Health Portal	Healthcare providers, researchers, patients/public	"Reviewed:" provided, but no precise update frequency given. Content published daily

## Discussion

This study assessed the quality of websites containing DHSs consumer health information for weight loss. With the increasingly popular use of DHSs and ease of web-based health information access [6, 7, 11, 12], this study helped to identify the overall quality of information patients may access on this topic. Our findings suggest the importance for healthcare providers to take an active role in understanding the types of websites typically accessed by patients in order to help them navigate (mis)information and refer them to high-quality resources.

Across 87 unique websites, most succeeded in describing the benefits of DHSs, and detailing and achieving their specified aims, while generally failing to describe the impact of treatment on overall quality of life, as well as the impact of a no treatment option. The highest-scoring websites were overwhelmingly categorized as health portals and government websites, while the majority of the lowest-scoring websites were commercial websites.

While to our knowledge, few studies that have investigated the quality of DHS consumer health information for weight loss have been published over the past 5 years, older studies and studies published on related topics can serve as comparative literature. In a 2014 study [22], researchers analysed the quality and comprehensiveness of online information related to weight loss. They concluded that web-based weight loss information was often of substandard quality because most comprehensive and high-quality websites ranked too low in the search results. Researchers attributed this to low search-engine optimization amongst medical, government, and university organization websites [22]. Previous studies have used the DISCERN instrument to assess web-based information surrounding chronic pain, gastrointestinal diseases, anxiety disorders, back pain, rheumatoid arthritis, and postherpetic neuralgia [23–28]. The quality of information found on these websites across all studies were moderate to poor; the vast majority of websites yielded by search results were commercial in nature. These studies also noted that higher-scoring websites tended to cite the research literature and provided a clear statement of purpose. High DISCERN instrument score variability across websites was also a common trend. All of these patterns similarly reflect what was observed in the present study.

### Strengths and limitations

One strength included the use of the DISCERN instrument, which has been found to be reliable and valid for assessing the quality of consumer health information on the internet. Conducting a pilot test standardized the application of the DISCERN instrument between individual reviewers. Another strength included searching in more than one country, which allowed for a more internationally representative sample of websites. This also allowed the results of this study to be more applicable to healthcare practitioners in multiple countries. Additionally, due to Google’s market share amongst search engines, conducting all searches using Google was likely representative of how the majority of patients seek web-based health information.

A notable limitation i that the internet is dynamic and constantly changing, thus the websites assessed in this study will not necessarily reflect information readily available to patients at another point in time. Additionally, conducting the pilot test with only three websites may not have been sufficient in standardizing assessments between each independent reviewer. As a result, this may have caused greater variation between DISCERN instrument scores for each website. Furthermore, the present study did not account for non-English language websites. Another limitation includes the fact that only six search terms were utilized across all regional searches. Therefore, it is very possible that a slight change in the wording of search terms could lead to variation in search results.

## Conclusion

The purpose of our study was to assess the quality of DHSs consumer health information for weight loss. High-scoring websites were largely health portals and government websites, while low-scoring websites were overwhelmingly commercial websites. Most websites described treatment benefits, and detailed and achieved their specified aims, but were lacking in their descriptions for overall impact on quality of life, and impact of a no treatment option. Large variability in DISCERN instrument scores exist between the assessed subset of websites, with a relatively equal distribution between high and low overall scores. Our findings suggest that the quality of websites being accessed by patients is likely of varying quality, thus healthcare providers need to be actively aware of this to guide patient information-seeking behaviour, as well as safe, effective, and evidence-informed decision making relating to DHSs use.

## Supplementary Information

**Additional file 1: Supplementary file 1** DISCERN Instrument Ratings for Eligible Websites.

## Data Availability

All relevant data are included in this manuscript.

## Abbreviation

DHSs: Dietary and herbal supplements

## References

[1] Hales CM, Carroll MD, Fryar CD, Ogden CL. Prevalence of obesity and severe obesity among adults: United States, 2017–2018 [Internet]. Centers for Disease Control and Prevention; 2020 [cited 2020 Sep 14]. Available from: https://www.cdc.gov/nchs/products/databriefs/db360.htm

[2] Childhood Obesity Facts [Internet]. Centers for disease control and prevention; 2019 [cited 2020 May 8]. Available from: https://www.cdc.gov/obesity/data/childhood.html

[3] Sarwer DB, Polonsky HM (2016). The psychosocial burden of obesity. Endocrinol Metab Clin.

[4] Barrea L, Altieri B, Polese B, De Conno B, Muscogiuri G, Colao A, Savastano S (2019). Nutritionist and obesity: brief overview on efficacy, safety, and drug interactions of the main weight-loss dietary supplements. Int J Obes Suppl.

[5] Dietary and Herbal Supplements [Internet]. National Center for Complementary and Integrative Health. U.S. Department of Health and Human Services; 2020 [cited 2020 Sep 14]. Available from: https://www.nccih.nih.gov/health/dietary-and-herbal-supplements

[6] Blanck HM, Serdula MK, Gillespie C, Galuska DA, Sharpe PA, Conway JM, Khan LK, Ainsworth BE (2007). Use of nonprescription dietary supplements for weight loss is common among Americans. J Am Diet Assoc.

[7] Is sports nutrition its own worst enemy? Nutr Bus J. 2014;19. https://www.newhope.com/news/sports-nutrition-its-own-worst-enemy.

[8] Natural Health Products [Internet]. Government of Canada; 2020 [cited 2020 Sep 14]. Available from: https://www.canada.ca/en/health-canada/services/drugs-health-products/natural-non-prescription.html

[9] Saldanha LG, Dwyer JT, Andrews KW, Bailey RL, Gahche JJ, Hardy CJ, Holden JM, Picciano MF, Roseland JM, Thomas PR, Wolf WR (2010). Online dietary supplement resources. J Am Diet Assoc.

[10] Wharton S, Bonder R, Jeffery A, Christensen RA. The safety and effectiveness of commonly marketed natural supplements for weight loss in populations with obesity: a critical review of the literature from 2006 to 2016. Critical reviews in food science and nutrition. 2019:1-710.1080/10408398.2019.1584873 10.1080/10408398.2019.1584873 30896252

[11] Internet/Broadband Fact Sheet [Internet]. Pew Research Center: Internet, Science Tech; 2019 [cited 2020 Sep 14]. Available from: https://www.pewresearch.org/internet/fact-sheet/internet-broadband/

[12] Fox S, Duggan M (2013). Health online. Pew Internet and American Life Project.

[13] Prescott C. Internet access – households and individuals, Great Britain: 2020 [Internet]. Office for National Statistics; 2020 [cited 2020 Sep 14]. Available from: https://www.ons.gov.uk/peoplepopulationandcommunity/householdcharacteristics/homeinternetandsocialmediausage/bulletins/internetaccesshouseholdsandindividuals/2020

[14] Diviani N, van den Putte B, Giani S, van Weert JC (2015). Low health literacy and evaluation of online health information: a systematic review of the literature. J Med Internet Res.

[15] Kalichman SC, Cherry C, White D, Kalichman MO, Detorio MA, Caliendo AM, Schinazi RF (2012). Use of dietary supplements among people living with HIV/AIDS is associated with vulnerability to medical misinformation on the internet. AIDS Res Ther.

[16] De Smet PA (2002). Herbal remedies. N Engl J Med.

[17] Dietary Supplements: What You Need to Know [Internet]. NIH Office of Dietary Supplements. U.S. Department of Health and Human Services; 2020 [cited 2020 Sep 14]. Available from: https://ods.od.nih.gov/HealthInformation/DS_WhatYouNeedToKnow.aspx

[18] The Value of Google Result Positioning [Internet]. Chitika Insights; 2013 [cited 2020 Sep 14]. Available from: http://info.chitika.com/uploads/4/9/2/1/49215843/chitikainsights-valueofgoogleresultspositioning.pdf

[19] Search Engine Market Share Worldwide [Internet]. StatCounter Global Stats; 2020 [cited 2020 Sep 14]. Available from: https://gs.statcounter.com/search-engine-market-share

[20] Charnock D, Shepperd S, Needham G, Gann R (1999). DISCERN: an instrument for judging the quality of written consumer health information on treatment choices. J Epidemiol Commun Health.

[21] FDA 101: Dietary Supplements [Internet]. U.S. Food and Drug Administration. FDA; 2015 [cited 2020 Sep 14]. Available from: https://www.fda.gov/consumers/consumer-updates/fda-101-dietary-supplements

[22] Modave F, Shokar NK, Peñaranda E, Nguyen N (2014). Analysis of the accuracy of weight loss information search engine results on the internet. Am J Public Health.

[23] Kaicker J, Debono VB, Dang W, Buckley N, Thabane L (2010). Assessment of the quality and variability of health information on chronic pain websites using the DISCERN instrument. BMC Med.

[24] Tangri V, Chande N. Quality of Internet-based information on gastrointestinal diseases. Can J Gastroenterol. 2011;25. doi: 10.1155/2011/345076. 10.1155/2011/345076 PMC3043011 21321681

[25] Ipser JC, Dewing S, Stein DJ (2007). A systematic review of the quality of information on the treatment of anxiety disorders on the internet. Curr Psychiatry Rep.

[26] Culver M, Chadwick A (2005). Internet information on rheumatoid arthritis: an evaluation. Musculoskeletal Care.

[27] Li L, Irvin E, Guzmán J, Bombardier C (2001). Surfing for back pain patients: the nature and quality of back pain information on the Internet. Spine.

[28] Hallingbye T, Serafini M (2011). Assessment of the quality of postherpetic neuralgia treatment information on the internet. J Pain.

