# Supplementary material for: Dietary and herbal supplements for weight loss: assessing the quality of patient information online
Source: Nutr J. 2021 Jul 27;20:72. doi: 10.1186/s12937-021-00729-x (PMC8317391; doi:10.1186/s12937-021-00729-x)
Supplement: Supplementary file 1 — Additional file 1: Supplementary file 1 DISCERN Instrument Ratings for Eligible Websites. [file 12937_2021_729_MOESM1_ESM.docx]

## Supplementary File 1: DISCERN Instrument Ratings for Eligible Websites

| **Section** | | **SECTION 1 Is the publication reliable?** | | | | | | | | **SECTION 2 How good is the quality of information on treatment choices?** | | | | | | | **SECTION 3 Overall Rating of the Publication** |  | |
| --- | --- | --- | --- | --- | --- | --- | --- | --- | --- | --- | --- | --- | --- | --- | --- | --- | --- | --- | --- |
| **DISCERN Question** | | **1. Are the aims clear?** | **2. Does it achieve its aims?** | **3. Is it relevant?** | **4. Is it clear what sources of information were used to compile the publication (other than the author or producer)?** | **5. Is it clear when the information used or reported in the publication was produced?** | **6. Is it balanced and unbiased?** | **7. Does it provide details of additional sources of support and information?** | **8. Does it refer to areas of uncertainty?** | **9. Does it describe how each treatment works?** | **10.Does it describe the benefits of each treatment?** | **11. Does it describe the risks of each treatment?** | **12. Does it describe what would happen if no treatment is used?** | **13. Does it describe how the treatment choices affect overall quality of life?** | **14. Is it clear that there may be more than one possible treatment choice?** | **15. Does it provide support for shared decision-making?** | **16. Based on the answers to all of the above questions, rate the overall quality of the publication as a source of information about treatment choices** | **Standard Deviation of Overall Score (Q16)** | **DISCERN Score (Sum of Q1-Q15)** |
| Very Well Fit | https://www.verywellfit.com/ | 3.50 | 5.00 | 4.50 | 5.00 | 5.00 | 5.00 | 5.00 | 5.00 | 2.50 | 5.00 | 5.00 | 2.00 | 5.00 | 5.00 | 5.00 | 5.00 | 0.00 | 67.50 |
| NIH Office of Dietary Supplements | [https://ods.od.nih.gov/](https://ods.od.nih.gov/factsheets/WeightLoss-HealthProfessional/) | 5.00 | 5.00 | 5.00 | 4.50 | 5.00 | 5.00 | 5.00 | 5.00 | 4.50 | 5.00 | 5.00 | 1.00 | 1.50 | 5.00 | 5.00 | 4.50 | 0.71 | 66.50 |
| Mayo Clinic | [https://www.mayoclinic.org/](https://www.mayoclinic.org/healthy-lifestyle/weight-loss/in-depth/weight-loss/art-20046409) | 5.00 | 5.00 | 5.00 | 3.00 | 5.00 | 5.00 | 4.50 | 5.00 | 3.50 | 5.00 | 3.50 | 3.50 | 3.50 | 5.00 | 5.00 | 4.50 | 0.71 | 66.50 |
| Drugs | [https://www.drugs.com/](https://www.drugs.com/article/prescription-weight-loss-drugs.html) | 5.00 | 5.00 | 5.00 | 4.50 | 5.00 | 5.00 | 5.00 | 5.00 | 3.50 | 5.00 | 5.00 | 1.00 | 2.00 | 5.00 | 5.00 | 4.50 | 0.71 | 66.00 |
| Healthline | [https://www.healthline.com/](https://www.healthline.com/nutrition/12-weight-loss-pills-reviewed) | 5.00 | 5.00 | 5.00 | 4.00 | 4.50 | 5.00 | 3.50 | 5.00 | 4.50 | 5.00 | 4.50 | 1.50 | 3.00 | 5.00 | 5.00 | 4.50 | 0.71 | 65.50 |
| WebMD | [https://www.webmd.com/](https://www.webmd.com/vitamins-and-supplements/herbal-remedies#1) | 5.00 | 5.00 | 4.50 | 3.00 | 5.00 | 5.00 | 5.00 | 5.00 | 3.50 | 5.00 | 5.00 | 1.50 | 3.00 | 5.00 | 5.00 | 4.00 | 0.00 | 65.50 |
| Examine | [https://examine.com/](https://examine.com/supplements/fat-loss/) | 5.00 | 5.00 | 4.50 | 4.50 | 5.00 | 5.00 | 5.00 | 5.00 | 4.50 | 5.00 | 5.00 | 1.00 | 1.50 | 4.50 | 4.50 | 4.50 | 0.71 | 65.00 |
| Medline Plus | [https://medlineplus.gov/](https://medlineplus.gov/ency/patientinstructions/000347.htm) | 5.00 | 5.00 | 4.50 | 3.50 | 4.50 | 5.00 | 5.00 | 5.00 | 2.00 | 5.00 | 4.50 | 1.00 | 5.00 | 5.00 | 5.00 | 3.50 | 0.71 | 65.00 |
| Cleveland Clinic | [https://my.clevelandclinic.org/](https://my.clevelandclinic.org/health/drugs/9469-over-the-counter-and-herbal-remedies-for-weight-loss) | 5.00 | 5.00 | 5.00 | 3.50 | 5.00 | 4.50 | 5.00 | 5.00 | 3.50 | 5.00 | 5.00 | 1.50 | 1.50 | 5.00 | 5.00 | 3.50 | 0.71 | 64.50 |
| Medical News Today | [https://www.medicalnewstoday.com/](https://www.medicalnewstoday.com/articles/320646) | 3.50 | 5.00 | 4.50 | 5.00 | 5.00 | 5.00 | 5.00 | 4.50 | 2.50 | 5.00 | 4.50 | 1.50 | 1.50 | 5.00 | 5.00 | 4.50 | 0.71 | 62.50 |
| Government of Canada | [https://www.canada.ca/](https://www.canada.ca/en/health-canada/services/drugs-medical-devices/safe-use-health-products-weight-loss.html) | 5.00 | 5.00 | 5.00 | 1.00 | 2.50 | 3.00 | 2.00 | 5.00 | 3.00 | 5.00 | 5.00 | 5.00 | 5.00 | 5.00 | 5.00 | 4.50 | 0.71 | 61.50 |
| Medscape | [https://www.medscape.com/](https://www.medscape.com/viewarticle/926081) | 5.00 | 5.00 | 4.50 | 4.50 | 5.00 | 5.00 | 4.50 | 5.00 | 2.50 | 5.00 | 5.00 | 1.00 | 1.00 | 5.00 | 3.00 | 4.00 | 0.00 | 61.00 |
| Nutrition House | [https://www.nutritionhouse.com/](https://www.nutritionhouse.com/AskTheExperts.aspx) | 5.00 | 4.50 | 4.50 | 4.50 | 3.00 | 4.50 | 5.00 | 5.00 | 3.50 | 4.50 | 4.50 | 1.00 | 1.00 | 5.00 | 5.00 | 4.50 | 0.71 | 60.50 |
| The Healthy | [https://www.thehealthy.com/](https://www.thehealthy.com/nutrition/vitamins/best-vitamins-weight-loss/) | 4.50 | 5.00 | 5.00 | 5.00 | 5.00 | 4.00 | 5.00 | 5.00 | 3.50 | 5.00 | 3.00 | 1.00 | 1.50 | 2.50 | 5.00 | 4.00 | 0.00 | 60.00 |
| Consumer Health Day | [https://consumer.healthday.com/](https://consumer.healthday.com/encyclopedia/holistic-medicine-25/mis-alternative-medicine-news-19/herbal-weight-loss-pills-648311.html) | 5.00 | 5.00 | 4.50 | 3.00 | 5.00 | 5.00 | 5.00 | 5.00 | 4.50 | 4.50 | 4.50 | 1.00 | 1.00 | 5.00 | 1.00 | 3.50 | 0.71 | 59.00 |
| Wexner Medical | [https://wexnermedical.osu.edu/](https://wexnermedical.osu.edu/blog/can-supplements-help-with-weight-loss) | 5.00 | 5.00 | 3.00 | 2.50 | 2.50 | 3.50 | 3.50 | 5.00 | 3.50 | 5.00 | 5.00 | 1.50 | 1.50 | 5.00 | 5.00 | 3.00 | 0.00 | 56.50 |
| Choice | [https://www.choice.com.au/](https://www.choice.com.au/health-and-body/diet-and-fitness/weight-loss/articles/weight-loss-pills) | 5.00 | 5.00 | 4.50 | 3.00 | 4.00 | 4.50 | 2.50 | 5.00 | 3.00 | 5.00 | 5.00 | 1.00 | 1.50 | 5.00 | 2.50 | 3.50 | 0.71 | 56.50 |
| Insider | [https://www.insider.com/](https://www.insider.com/do-diet-pills-work) | 3.50 | 4.50 | 4.50 | 3.50 | 3.00 | 5.00 | 2.50 | 5.00 | 1.50 | 5.00 | 4.50 | 1.50 | 1.50 | 5.00 | 4.50 | 3.50 | 0.71 | 55.00 |
| Medicine Net | [https://www.medicinenet.com/](https://www.medicinenet.com/script/main/art.asp?articlekey=42604) | 5.00 | 5.00 | 5.00 | 1.50 | 2.50 | 3.50 | 1.50 | 5.00 | 3.50 | 5.00 | 5.00 | 1.00 | 1.50 | 5.00 | 4.50 | 3.50 | 0.71 | 54.50 |
| Nutrain Ingredients | [https://www.nutraingredients.com/](https://www.nutraingredients.com/Article/2020/02/20/Evidence-for-weight-loss-herbal-supplements-branded-insufficient) | 5.00 | 5.00 | 5.00 | 5.00 | 3.00 | 5.00 | 4.50 | 5.00 | 3.00 | 3.50 | 2.50 | 1.00 | 1.00 | 3.00 | 3.00 | 3.50 | 0.71 | 54.50 |
| Empower Your Health | [https://www.empoweryourhealth.org/](https://www.empoweryourhealth.org/magazine/vol4_issue2/Supplements-and-Weight-Loss-Are-There-Natural-Ways-to-Lose-Weight) | 5.00 | 5.00 | 3.50 | 1.00 | 1.50 | 3.50 | 4.00 | 4.50 | 3.00 | 5.00 | 3.00 | 1.00 | 2.50 | 5.00 | 5.00 | 3.00 | 0.00 | 52.50 |
| The Cut | [https://www.thecut.com/](https://www.thecut.com/2014/01/i-tried-7-natural-weight-loss-supplements.html) | 3.50 | 4.00 | 3.50 | 3.00 | 3.50 | 4.50 | 2.50 | 4.50 | 2.50 | 5.00 | 5.00 | 1.50 | 1.50 | 3.00 | 4.50 | 3.50 | 0.71 | 52.00 |
| Body Nutrition | [https://bodynutrition.org/](https://bodynutrition.org/weight-loss-pills/) | 4.50 | 5.00 | 4.00 | 2.50 | 3.50 | 3.50 | 2.50 | 2.50 | 3.00 | 5.00 | 3.50 | 1.00 | 1.50 | 5.00 | 5.00 | 3.50 | 0.71 | 52.00 |
| Herbal Magic | [https://www.herbalmagic.ca/](https://www.herbalmagic.ca/products/3-month-weight-loss-plan) | 5.00 | 5.00 | 5.00 | 5.00 | 3.00 | 2.50 | 5.00 | 1.00 | 3.00 | 5.00 | 2.00 | 1.00 | 3.00 | 2.00 | 4.50 | 3.50 | 0.71 | 52.00 |
| Future's Recovery Healthcare | [https://futuresrecoveryhealthcare.com/](https://futuresrecoveryhealthcare.com/knowledge-center/harmful-effects-diet-pills-supplements/) | 5.00 | 5.00 | 4.00 | 1.50 | 3.50 | 2.50 | 2.00 | 3.00 | 2.50 | 3.50 | 5.00 | 1.00 | 3.50 | 5.00 | 4.50 | 3.00 | 0.00 | 51.50 |
| Legion Athletics | [https://legionathletics.com/](https://legionathletics.com/the-best-fat-loss-supplements/) | 5.00 | 5.00 | 3.50 | 4.50 | 2.50 | 3.00 | 5.00 | 3.50 | 4.50 | 4.50 | 3.00 | 1.00 | 1.50 | 2.50 | 2.50 | 3.00 | 0.00 | 51.50 |
| Draxe | [https://draxe.com/](https://draxe.com/health/fat-burners/) | 3.50 | 5.00 | 3.50 | 3.00 | 3.00 | 4.50 | 2.50 | 2.50 | 3.50 | 5.00 | 2.50 | 5.00 | 1.00 | 2.50 | 4.50 | 3.00 | 0.00 | 51.50 |
| LifeHack | [https://www.lifehack.org/](https://www.lifehack.org/853736/natural-weight-loss-supplements) | 5.00 | 5.00 | 3.50 | 3.50 | 3.00 | 2.50 | 3.50 | 1.00 | 4.00 | 5.00 | 1.00 | 5.00 | 2.00 | 4.50 | 2.50 | 3.00 | 0.00 | 51.00 |
| OpenFit | [https://www.openfit.com/](https://www.openfit.com/herbs-for-weight-loss) | 3.50 | 5.00 | 4.00 | 3.50 | 3.00 | 4.50 | 4.50 | 3.00 | 2.50 | 5.00 | 2.50 | 1.00 | 1.00 | 3.00 | 5.00 | 3.50 | 0.71 | 51.00 |
| Remedy Review | [https://www.remedyreview.com/](https://www.remedyreview.com/sport/herbs-for-weight-loss/) | 5.00 | 5.00 | 3.00 | 3.00 | 3.00 | 4.50 | 2.50 | 2.50 | 3.50 | 5.00 | 3.50 | 1.00 | 1.00 | 2.50 | 5.00 | 3.00 | 0.00 | 50.00 |
| Mind Body Green | [https://www.mindbodygreen.com/](https://www.mindbodygreen.com/0-4866/3-Herbs-for-Weight-Loss-Support.html) | 5.00 | 5.00 | 4.50 | 3.00 | 3.00 | 3.50 | 2.50 | 2.50 | 3.50 | 5.00 | 2.50 | 1.00 | 1.50 | 2.50 | 4.50 | 3.50 | 0.71 | 49.50 |
| Health | [https://www.health.com/](https://www.health.com/weight-loss/pros-cons-fitness-supplements) | 4.50 | 5.00 | 3.00 | 3.00 | 3.50 | 4.50 | 2.50 | 5.00 | 1.50 | 3.00 | 3.00 | 1.00 | 1.50 | 4.50 | 3.50 | 3.00 | 0.00 | 49.00 |
| Global Healing | [https://globalhealing.com/](https://globalhealing.com/natural-health/top-herbs-for-weight-loss/) | 5.00 | 5.00 | 3.50 | 3.50 | 5.00 | 2.50 | 5.00 | 2.00 | 2.50 | 5.00 | 1.50 | 1.50 | 1.00 | 2.50 | 3.00 | 3.00 | 0.00 | 48.50 |
| Coach Nine | [https://coach.nine.com.au/](https://coach.nine.com.au/fitness/fat-burning-supplements/0c437491-6be5-4112-915a-267e41ba2e46) | 3.50 | 4.50 | 4.50 | 3.00 | 3.00 | 5.00 | 2.50 | 5.00 | 2.50 | 5.00 | 4.50 | 1.50 | 1.50 | 1.50 | 1.00 | 3.50 | 0.71 | 48.50 |
| Natural Grocers | [https://www.naturalgrocers.com/](https://www.naturalgrocers.com/article/weight-loss-healthy-way) | 5.00 | 4.50 | 4.50 | 3.00 | 3.00 | 3.00 | 5.00 | 1.50 | 3.00 | 5.00 | 1.50 | 1.50 | 1.50 | 2.50 | 2.50 | 3.00 | 0.00 | 47.00 |
| Natural Health Courses | [https://naturalhealthcourses.com/](https://naturalhealthcourses.com/2015/07/holistic-approach-weight-loss/) | 5.00 | 5.00 | 4.00 | 2.50 | 4.50 | 3.00 | 4.50 | 1.00 | 1.50 | 5.00 | 1.50 | 1.00 | 3.00 | 3.00 | 2.50 | 2.50 | 0.71 | 47.00 |
| Holland and Barrett | https://www.hollandandbarrett.com/ | 4.50 | 5.00 | 4.00 | 2.50 | 2.50 | 3.00 | 4.00 | 1.50 | 2.50 | 5.00 | 2.50 | 1.00 | 1.00 | 2.50 | 4.50 | 3.00 | 0.00 | 46.00 |
| Shape | [https://www.shape.com/](https://www.shape.com/weight-loss/tips-plans/natural-weight-loss-tips) | 2.50 | 3.00 | 4.50 | 3.00 | 1.50 | 4.50 | 2.50 | 5.00 | 2.50 | 3.50 | 3.50 | 2.50 | 1.50 | 4.50 | 1.00 | 3.50 | 0.71 | 45.50 |
| National University of Health Sciences | [https://blog.nuhs.edu/](https://blog.nuhs.edu/the-future-of-integrative-health/weight-loss-tips-from-a-traditional-chinese-medicine-expert) | 5.00 | 5.00 | 4.50 | 2.50 | 3.00 | 3.50 | 2.50 | 1.00 | 1.50 | 5.00 | 3.00 | 1.00 | 1.50 | 3.50 | 3.00 | 2.50 | 0.71 | 45.50 |
| Botanica Health | [https://botanicahealth.com/](https://botanicahealth.com/blog/best-herbs-weight-loss/) | 5.00 | 5.00 | 3.00 | 3.00 | 4.50 | 3.00 | 5.00 | 1.00 | 2.50 | 5.00 | 1.00 | 1.00 | 1.00 | 2.00 | 3.50 | 3.00 | 0.00 | 45.50 |
| RenueRX | [https://renuerx.com/](https://renuerx.com/3-metabolism-boosting-pills-supplements-to-help-weight-loss/) | 4.50 | 5.00 | 3.50 | 3.00 | 2.00 | 3.50 | 2.50 | 2.50 | 1.50 | 5.00 | 1.00 | 1.00 | 1.50 | 5.00 | 3.00 | 2.50 | 0.71 | 44.50 |
| Style Craze | [https://www.stylecraze.com/](https://www.stylecraze.com/articles/home-remedies-for-fat-burn/) | 5.00 | 5.00 | 4.00 | 3.00 | 3.00 | 3.50 | 2.50 | 1.00 | 1.50 | 5.00 | 1.00 | 1.50 | 1.00 | 4.50 | 3.00 | 2.50 | 0.71 | 44.50 |
| Fusion Health | [https://www.fusionhealth.com.au/](https://www.fusionhealth.com.au/taxonomy/term/4956) | 5.00 | 5.00 | 4.50 | 3.00 | 1.50 | 2.50 | 3.50 | 1.00 | 4.00 | 5.00 | 1.50 | 1.50 | 1.50 | 1.50 | 3.50 | 3.00 | 0.00 | 44.50 |
| Indigo Herbs | [https://www.indigo-herbs.co.uk/](https://www.indigo-herbs.co.uk/natural-health-guide/benefits/weight-loss) | 5.00 | 5.00 | 4.50 | 1.50 | 1.50 | 2.50 | 1.50 | 1.00 | 4.50 | 5.00 | 2.50 | 1.00 | 1.50 | 2.50 | 3.50 | 3.00 | 0.00 | 43.00 |
| Prevention | [https://www.prevention.com/](https://www.prevention.com/weight-loss/a20493821/herbs-and-spices-for-weight-loss/) | 3.50 | 3.50 | 3.00 | 3.00 | 3.00 | 4.50 | 1.50 | 1.50 | 2.00 | 5.00 | 1.00 | 1.00 | 1.50 | 5.00 | 2.50 | 3.00 | 0.00 | 41.50 |
| CVS | [https://www.cvs.com/](https://www.cvs.com/shop/diet-nutrition/weight-loss/weight-loss-supplements) | 5.00 | 3.50 | 5.00 | 1.00 | 1.50 | 2.50 | 1.00 | 1.00 | 1.50 | 5.00 | 2.50 | 1.00 | 1.00 | 5.00 | 4.50 | 2.00 | 0.00 | 41.00 |
| Natural Foods Market Online | [https://nfmonline.com/](https://nfmonline.com/herbs-for-weight-loss) | 5.00 | 4.50 | 3.50 | 4.50 | 1.50 | 2.50 | 4.50 | 1.00 | 1.50 | 5.00 | 1.00 | 1.00 | 1.50 | 3.00 | 1.00 | 3.50 | 0.71 | 41.00 |
| PatchMD | [https://www.patchmd.com/](https://www.patchmd.com/weight-loss-simplified-the-best-bariatric-vitamins-to-aid-your-weight-loss-b-70.html) | 2.50 | 5.00 | 3.50 | 3.00 | 3.00 | 1.50 | 2.50 | 3.00 | 2.50 | 5.00 | 1.50 | 1.00 | 1.00 | 2.50 | 2.50 | 2.00 | 0.00 | 40.00 |
| Women's Health Magazine | [https://www.womenshealthmag.com/uk/](https://www.womenshealthmag.com/uk/health/conditions/a703721/25-of-the-best-supplements-pills-worth-popping/) | 4.50 | 3.50 | 3.00 | 1.50 | 2.50 | 1.50 | 1.00 | 3.00 | 2.00 | 5.00 | 2.50 | 1.00 | 1.50 | 3.50 | 3.00 | 3.00 | 0.00 | 39.00 |
| My Protein | [https://www.myprotein.com/](https://www.myprotein.com/nutrition/weight-management/weight-loss-supplements.list) | 3.50 | 4.50 | 3.00 | 1.50 | 2.50 | 1.50 | 5.00 | 1.00 | 2.50 | 5.00 | 1.50 | 1.00 | 1.50 | 2.00 | 3.00 | 2.00 | 0.00 | 39.00 |
| NDTV | https://doctor.ndtv.com/ | 4.00 | 4.50 | 3.50 | 1.00 | 2.50 | 1.50 | 1.00 | 1.00 | 1.50 | 5.00 | 2.50 | 1.00 | 1.50 | 5.00 | 3.50 | 2.50 | 0.71 | 39.00 |
| Lose Weight Loss | [http://www.loseweightloss.net/](http://www.loseweightloss.net/blog/herbs-and-spices-for-weight-loss/) | 5.00 | 5.00 | 4.00 | 2.50 | 2.50 | 2.50 | 2.50 | 1.00 | 1.50 | 5.00 | 1.00 | 1.00 | 1.00 | 3.00 | 1.50 | 2.00 | 0.00 | 39.00 |
| Bulk Nutrients | [https://www.bulknutrients.com.au/](https://www.bulknutrients.com.au/categories/weightloss/) | 4.50 | 5.00 | 4.50 | 3.00 | 1.50 | 2.00 | 2.50 | 1.00 | 4.50 | 5.00 | 1.00 | 1.00 | 1.00 | 1.50 | 1.00 | 2.50 | 0.71 | 39.00 |
| Times of India | [https://timesofindia.indiatimes.com/](https://timesofindia.indiatimes.com/life-style/health-fitness/weight-loss/8-herbs-that-can-give-your-weight-loss-plan-a-boost/articleshow/63730601.cms) | 3.50 | 5.00 | 3.00 | 1.50 | 2.50 | 3.00 | 1.50 | 1.00 | 1.50 | 5.00 | 1.00 | 1.00 | 1.00 | 5.00 | 3.00 | 2.00 | 0.00 | 38.50 |
| Botanic Choice | [https://www.botanicchoice.com/](https://www.botanicchoice.com/blog/diet-and-recipes/top-10-best-natural-herbs-for-weight-loss/) | 4.50 | 4.50 | 3.00 | 1.50 | 2.50 | 1.50 | 5.00 | 1.00 | 1.50 | 4.50 | 1.00 | 1.00 | 1.00 | 2.50 | 3.50 | 2.50 | 0.71 | 38.50 |
| GNC | [https://www.gnc.com/](https://www.gnc.com/weight-loss-diet/how-to-supplement-tips-for-healthy-weight-loss-and-appetite-control.html) | 5.00 | 5.00 | 4.00 | 1.00 | 1.50 | 1.50 | 2.00 | 1.00 | 2.50 | 5.00 | 1.50 | 1.00 | 1.50 | 2.50 | 3.00 | 1.50 | 0.71 | 38.00 |
| Brighter Day Foods | [https://brighterdayfoods.com/](https://brighterdayfoods.com/herbs-for-weight-loss) | 3.00 | 4.00 | 3.00 | 4.50 | 1.50 | 2.50 | 4.00 | 1.50 | 3.00 | 4.50 | 1.00 | 1.00 | 1.00 | 2.50 | 1.00 | 2.00 | 0.00 | 38.00 |
| Australian Sports Nutrition | [https://www.australiansportsnutrition.com.au/](https://www.australiansportsnutrition.com.au/shop-by-category/fat-burning.html) | 5.00 | 3.50 | 3.50 | 1.50 | 3.00 | 1.50 | 5.00 | 1.00 | 3.50 | 5.00 | 1.00 | 1.00 | 1.00 | 1.50 | 1.00 | 2.50 | 0.71 | 38.00 |
| Men's Journal | [https://www.mensjournal.com/](https://www.mensjournal.com/food-drink/10-best-supplements-weight-loss/) | 1.50 | 3.00 | 2.50 | 3.00 | 1.50 | 3.50 | 2.50 | 1.50 | 3.50 | 5.00 | 1.50 | 1.00 | 1.50 | 2.50 | 3.50 | 2.50 | 0.71 | 37.50 |
| Active | [https://www.active.com/](https://www.active.com/nutrition/articles/top-5-herbs-for-weight-loss) | 4.50 | 5.00 | 4.50 | 1.00 | 1.50 | 1.50 | 1.00 | 1.00 | 1.50 | 5.00 | 3.00 | 1.00 | 1.00 | 3.00 | 3.00 | 2.50 | 0.71 | 37.50 |
| Craig Lewis Fitness | [https://craiglewisfitness.com/](https://craiglewisfitness.com/best-herbs-for-weight-loss-and-detox/) | 5.00 | 4.50 | 3.00 | 2.50 | 3.00 | 2.50 | 2.50 | 1.00 | 1.00 | 5.00 | 1.00 | 1.00 | 1.00 | 2.50 | 1.00 | 1.50 | 0.71 | 36.50 |
| PHD | [https://www.phd.com/](https://www.phd.com/perform-smart/fat-burners-101/) | 5.00 | 5.00 | 3.50 | 1.50 | 2.50 | 2.00 | 1.00 | 1.50 | 3.50 | 5.00 | 1.50 | 1.00 | 1.00 | 1.50 | 1.00 | 2.00 | 0.00 | 36.50 |
| True Protein | [https://www.trueprotein.com.au/](https://www.trueprotein.com.au/pages/diet-weight-loss-supplements) | 5.00 | 4.50 | 5.00 | 1.00 | 1.50 | 1.00 | 1.00 | 3.00 | 1.50 | 5.00 | 1.00 | 1.00 | 1.00 | 2.50 | 2.50 | 2.00 | 0.00 | 36.50 |
| Feel Good Natural | [https://feelgoodnatural.com/](https://feelgoodnatural.com/product-category/weight-loss/) | 4.50 | 4.50 | 4.00 | 1.00 | 1.50 | 1.50 | 1.00 | 1.00 | 1.50 | 4.50 | 2.00 | 1.00 | 1.00 | 2.50 | 3.50 | 1.50 | 0.71 | 35.00 |
| Family Living Today | [https://familylivingtoday.com/](https://familylivingtoday.com/best-weight-loss-supplements-and-diet-pills/) | 5.00 | 5.00 | 2.50 | 1.00 | 2.50 | 1.50 | 1.50 | 1.00 | 1.50 | 5.00 | 1.50 | 1.00 | 1.50 | 1.00 | 3.00 | 1.50 | 0.71 | 34.50 |
| NowFoods | [https://www.nowfoods.com/](https://www.nowfoods.com/now/nowledge/more-about-weight-management-supplements) | 3.00 | 5.00 | 5.00 | 1.00 | 1.50 | 1.50 | 1.00 | 1.00 | 2.50 | 4.50 | 1.00 | 1.00 | 1.00 | 2.50 | 3.00 | 1.50 | 0.71 | 34.50 |
| Carusos Natural Health | [https://carusosnaturalhealth.com.au/](https://carusosnaturalhealth.com.au/articles/how-to-lose-weight/) | 5.00 | 4.50 | 4.50 | 1.00 | 1.00 | 1.50 | 1.00 | 1.00 | 1.00 | 5.00 | 1.50 | 1.00 | 1.50 | 2.50 | 2.50 | 2.00 | 0.00 | 34.50 |
| Boots | [https://www.boots.com/](https://www.boots.com/health-pharmacy/lifestyle-wellbeing/weightloss) | 5.00 | 4.50 | 5.00 | 1.00 | 1.50 | 1.50 | 1.00 | 1.00 | 1.00 | 5.00 | 1.00 | 1.00 | 1.00 | 1.50 | 3.00 | 1.50 | 0.71 | 34.00 |
| The Natural Way | [https://www.thenaturalway.com.au](https://www.thenaturalway.com.au/)/ | 5.00 | 4.50 | 3.50 | 1.00 | 1.00 | 1.00 | 1.00 | 1.00 | 3.50 | 5.00 | 2.50 | 1.00 | 1.00 | 1.50 | 1.50 | 2.00 | 0.00 | 34.00 |
| Bodybuilding | [https://www.bodybuilding.com/](https://www.bodybuilding.com/store/best-fat-burner-supplements.html) | 3.50 | 4.50 | 3.50 | 1.00 | 1.50 | 1.50 | 1.00 | 1.00 | 2.00 | 5.00 | 1.50 | 1.00 | 1.00 | 2.50 | 3.00 | 1.50 | 0.71 | 33.50 |
| Bulk Powders | [https://www.bulkpowders.co.uk/](https://www.bulkpowders.co.uk/weight-loss-goal) | 3.50 | 5.00 | 2.50 | 1.00 | 1.50 | 1.50 | 1.00 | 3.00 | 3.50 | 5.00 | 1.50 | 1.00 | 1.00 | 1.00 | 1.50 | 2.00 | 0.00 | 33.50 |
| Express | [https://www.express.co.uk/](https://www.express.co.uk/life-style/health/1258801/best-supplements-weight-loss-caffeine-green-tea-extract-protein-powder) | 3.50 | 3.50 | 3.00 | 2.50 | 2.50 | 3.00 | 1.50 | 1.00 | 2.00 | 4.50 | 1.00 | 1.00 | 1.50 | 2.00 | 1.00 | 1.50 | 0.71 | 33.50 |
| Digital Welt | [https://www.digitalwelt.org/](https://www.digitalwelt.org/en/lifestyle/herbs/natural-herbs-for-weight-loss) | 3.50 | 4.50 | 3.50 | 1.00 | 1.00 | 1.50 | 1.00 | 1.00 | 1.00 | 4.50 | 3.00 | 1.00 | 1.00 | 2.50 | 3.00 | 2.50 | 0.71 | 33.00 |
| Nature's Best | [https://www.naturesbest.co.uk/](https://www.naturesbest.co.uk/weight-loss/) | 4.50 | 5.00 | 4.50 | 1.00 | 1.50 | 1.50 | 1.00 | 1.00 | 2.50 | 5.00 | 1.00 | 1.00 | 1.00 | 1.50 | 1.00 | 1.50 | 0.71 | 33.00 |
| Priceline | [https://www.priceline.com.au/](https://www.priceline.com.au/diet-and-nutrition/diet-and-weight-management/supplements) | 4.00 | 3.50 | 3.50 | 1.00 | 1.50 | 1.50 | 1.00 | 1.00 | 1.50 | 5.00 | 1.00 | 1.00 | 1.00 | 3.00 | 2.50 | 1.50 | 0.71 | 32.00 |
| Body and Soul | [https://www.bodyandsoul.com.au/](https://www.bodyandsoul.com.au/diet/natural-aids-to-weight-loss/news-story/96bbdc8e68a959d43c890b26339c37bf) | 5.00 | 3.50 | 3.00 | 1.00 | 2.50 | 1.50 | 1.00 | 1.00 | 1.00 | 5.00 | 1.00 | 1.00 | 1.00 | 1.50 | 2.50 | 1.50 | 0.71 | 31.50 |
| Pharmacy Online | [https://www.pharmacyonline.com.au/](https://www.pharmacyonline.com.au/weight-loss-fitness/weight-management/weight-loss-supplements) | 3.50 | 3.50 | 4.50 | 1.00 | 1.00 | 1.50 | 1.00 | 1.50 | 1.00 | 4.50 | 1.00 | 1.00 | 1.00 | 3.00 | 2.50 | 2.00 | 0.00 | 31.50 |
| Aus Natural Care | [https://www.ausnaturalcare.com.au/](https://www.ausnaturalcare.com.au/vitamins/Weight-Loss) | 5.00 | 3.50 | 3.00 | 1.00 | 1.50 | 1.00 | 1.00 | 1.00 | 2.50 | 5.00 | 1.50 | 1.00 | 1.00 | 1.00 | 2.50 | 1.50 | 0.71 | 31.50 |
| Shoppers Drug Mart | https://www1.shoppersdrugmart.ca/ | 4.50 | 3.50 | 3.00 | 1.00 | 1.00 | 2.50 | 1.00 | 1.00 | 1.00 | 3.00 | 1.00 | 1.00 | 1.00 | 3.50 | 3.00 | 1.50 | 0.71 | 31.00 |
| Go Healthy | [https://gohealthy.co.nz/](https://gohealthy.co.nz/products/weight-management/) | 4.50 | 3.50 | 4.50 | 1.00 | 1.50 | 1.50 | 1.00 | 1.00 | 1.00 | 4.50 | 1.50 | 1.00 | 1.00 | 1.00 | 2.50 | 1.50 | 0.71 | 31.00 |
| Chemist Warehouse | [https://www.chemistwarehouse.com.au/](https://www.chemistwarehouse.com.au/shop-online/517/weight-loss) | 3.00 | 2.50 | 3.50 | 1.00 | 1.00 | 1.50 | 1.00 | 1.00 | 2.50 | 4.50 | 3.00 | 1.00 | 1.00 | 2.50 | 1.50 | 1.50 | 0.71 | 30.50 |
| My Nutricentre | [https://www.mynutricentre.com](https://www.mynutricentre.com/)/ | 3.00 | 5.00 | 4.00 | 1.00 | 1.50 | 1.00 | 1.00 | 1.00 | 2.50 | 4.50 | 1.00 | 1.00 | 1.00 | 1.50 | 1.00 | 1.50 | 0.71 | 30.00 |
| Nutrition Warehouse | [https://www.nutritionwarehouse.com.au/](https://www.nutritionwarehouse.com.au/top-10-fat-burners.html) | 5.00 | 2.50 | 3.50 | 1.00 | 1.50 | 1.50 | 1.00 | 1.00 | 2.50 | 5.00 | 1.00 | 1.00 | 1.00 | 1.00 | 1.00 | 1.50 | 0.71 | 29.50 |
| My Vitamins | [https://www.myvitamins.com/](https://www.myvitamins.com/your-needs/weight-loss-supplements.list) | 3.50 | 5.00 | 3.00 | 1.00 | 1.50 | 1.00 | 1.00 | 1.00 | 1.50 | 5.00 | 1.00 | 1.00 | 1.00 | 1.50 | 1.00 | 1.50 | 0.71 | 29.00 |
| Healthy Being | [https://www.healthybeing.com.au/](https://www.healthybeing.com.au/natural-weight-loss/) | 4.50 | 4.50 | 3.00 | 1.00 | 1.50 | 1.00 | 1.00 | 1.00 | 1.00 | 5.00 | 1.00 | 1.00 | 1.00 | 1.50 | 1.00 | 1.50 | 0.71 | 29.00 |
| Supreme Natural Health | [https://supremenaturalhealth.com.au/](https://supremenaturalhealth.com.au/weightloss/) | 4.50 | 4.00 | 3.50 | 1.00 | 1.50 | 1.00 | 1.00 | 1.00 | 1.50 | 4.50 | 1.00 | 1.00 | 1.50 | 1.00 | 1.00 | 1.50 | 0.71 | 29.00 |
| Elite Supps | [https://www.elitesupps.com.au/](https://www.elitesupps.com.au/collections/weight-loss-supplements) | 3.50 | 3.50 | 3.50 | 1.00 | 1.50 | 1.00 | 1.00 | 1.00 | 1.50 | 5.00 | 1.50 | 1.00 | 1.00 | 1.00 | 1.00 | 1.50 | 0.71 | 28.00 |
| **TOTAL Means** | | 4.41 | 4.56 | 3.93 | 2.41 | 2.68 | 2.89 | 2.71 | 2.44 | 2.51 | 4.82 | 2.47 | 1.26 | 1.51 | 3.14 | 3.07 | 2.72 | 0.45 | 44.80 |
| **TOTAL Standard Deviations** | | 0.79 | 0.66 | 0.75 | 1.33 | 1.27 | 1.40 | 1.60 | 1.72 | 1.03 | 0.42 | 1.50 | 0.80 | 0.88 | 1.43 | 1.42 | 0.99 | 0.34 | 11.53 |
